# Causes of Pruritus in Patients Treated With Immune Checkpoint Inhibitors for Melanomas or Skin Carcinomas

**DOI:** 10.3389/fmed.2021.632683

**Published:** 2021-02-09

**Authors:** Nadia Salinas, Emmanuel Nowak, Maxime Etienne, Delphine Legoupil, Maxime Fouchard, Emilie Brenaut, Laurent Misery

**Affiliations:** ^1^Department of Dermatology, University Hospital of Brest, Brest, France; ^2^Université de Brest, LIEN, Brest, France; ^3^INSERM CIC 1412, University Hospital of Brest, Brest, France

**Keywords:** prurit, itch, survival, melanoma, squamous cell carcinoma, immune checkpoint inhibitors

## Abstract

**Background:** Pruritus is a frequent adverse event during the use of immune checkpoint inhibitors (ICIs), with a frequency estimated to be between 11 and 47%. The underlying causes remain poorly understood.

**Objectives:** The main goal was to search for putative causes of pruritus occurring in patients treated with ICIs for melanomas and cutaneous carcinomas. Other objectives were to assess the association between the occurrence of pruritus and survival and between the occurrence of pruritus and other adverse events.

**Methods:** A monocentric retrospective descriptive study was performed using data for patients treated with ICIs (nivolumab, pembrolizumab, ipilimumab, and cemiplimab) between August 2010 and November 2019.

**Results:** A total of 181 patients were included (mean age: 69 years). Pruritus was reported by 25 patients (13.8%). We were able to determine three subgroups of pruritus causes under ICI use: pruritus directly related to immunotherapy, pruritus indirectly related through other pruritus-inducing side effects and pruritus unrelated to ICIs. In 6/25 patients, no more specific cause of pruritus was found at the onset of pruritus or in their backgrounds, other than ICI use.

**Limitations:** The study has some limitations due to unicentric and retrospective design.

**Conclusion:** Pruritus was found in 25/181 patients in this series; only in 6/25 patients no potential cause other than ICI could be found, and pruritus was not associated with differences in survival.

## Introduction

Immune checkpoint inhibitors (ICIs) are commonly used in the therapeutic arsenal of metastatic melanoma, Merkel cell carcinoma and cutaneous squamous cell carcinoma due to their inhibitory effects on cytotoxic T-lymphocyte-associated protein 4 (anti-CTLA4) or anti-programmed death-1 (anti-PD1). Ipilimumab (anti-CTLA4), pembrolizumab and nivolumab (PD1 inhibitors) are the available ICIs used in melanoma for ipilimumab since 2014, for pembrolizumab and nivolumab since 2015. Advanced cutaneous squamous cell carcinoma has also been treated with ICIs since 2018, including cemiplimab (anti-PD1) (1–4). These treatments can induce numerous cutaneous and non-cutaneous adverse effects that are mainly due to their immunological effects (5–9). The most frequent adverse events are dysthyroidism, autoimmune hepatitis, colitis, and skin disorders (10–13). Among those, pruritus is frequently reported as a side effect of these treatments (14, 15), with an estimated incidence between 11 and 47% (16, 17). Pruritus can deeply affect the patient's quality of life and may lead to treatment discontinuation. However, ICI-related pruritus has been poorly studied to date and is not well-understood. In the literature, data on the presence and characteristics of pruritus in patients treated with ICIs have been provided, but without analyzing the causes of the pruritus (18). Indeed, it is not known whether the occurrence of pruritus is related to direct or indirect effects of ICIs. Moreover, some authors report a correlation between the occurrence of some cutaneous adverse events (but not pruritus) while taking ICIs and survival (19–23). The principal aim of our study was to analyse the putative causes of pruritus occurring without any skin lesions in patients treated with ICIs for melanomas and cutaneous carcinomas. Other objectives were to assess the association between the occurrence of pruritus and survival and between other adverse events and pruritus.

## Methods

A monocentric, retrospective and descriptive study was performed on patients treated with ICIs (nivolumab, pembrolizumab, ipilimumab, or cemiplimab) in the Department of Dermatology of the University Hospital of Brest and in the associated Department in the Hospital of Landerneau. The study was approved by the ethics committee of Brest (B2019CE.19) and was registered at clinicaltrials.gov (NCT04365244).

The inclusion criteria were as follows: age 18 years and older, treated with ICIs (nivolumab, pembrolizumab, ipilimumab, and cemiplimab) for melanoma, squamous cell carcinoma or Merkel cell carcinoma and not having formulated any opposition. The exclusion criteria were as follows: age under 18 years, adults not legally competent, presence of pruritus at inclusion, and participation refusal.

After assessing the inclusion and non-inclusion criteria, an information letter with a non-opposition form was sent to the patients. The information collected from clinical records included demographic data, history of the cancer and treatment, date of onset of pruritus and localization, pruritus characteristics, treatments, putative etiologies of pruritus, date and type of other adverse events, date of treatment discontinuation, date of illness progression, date of death, and date of loss to follow up.

The main objective was to determine the putative causes of pruritus.

The secondary objectives were to determine the presence of other side effects, to analyse survival according to the presence of pruritus and to determine whether the occurrence of pruritus is linked to other adverse events.

We conducted a descriptive analysis of the data. Quantitative data are described by their size, mean, standard deviation, median, extreme values (minimum and maximum), and number of missing data. Qualitative data are described by their distributions in terms of numbers and percentages and the number of missing data. We used Excel for descriptive analyses.

Overall survival (OS) was analyzed using a multivariable Cox model with a time-dependent covariate to examine the association between pruritus onset and survival. Pruritus onset was considered a dichotomous (present/absent) time-dependent covariate without transition back from a state of present to a state of absent. Pruritus exposure also declined according to its origin: immunotherapy-induced or systemic, leading to two dichotomous time-dependent covariates without transition back from a state of present to a state of absent. Hazard ratios (HRs) were calculated with their 95% confidence intervals (95% CIs) using the PHREG procedure (SAS software, version 9.4).

## Results

### Characteristics of the Population

One hundred and eighty-one patients were included (175 melanoma and six squamous cell carcinoma cases). Demographic data are presented in Table 1. In spite of our small numbers of patients when we compared patients without any pruritus to those who experienced pruritus and found significant differences in patient characteristics with regard to vitiligo and dysthyroidism (Table 1).

**Table 1:** Characteristics of patients and tumors.

	**All patients (*n* = 181)**	**Patients with pruritus (*n* = 25)**	**Patients without pruritus (*n* = 156)**	***p***
**SEX**, ***n*** **(%)**				
Men	92 (50.8)	12 (48.0)	80 (51.2)	0.83
Women	89 (49.2)	13 (52.0)	76 (48.7)	0.83
**AGE**				
Mean	69	73	69	
Median	72	76	72	
Range	27–97	41–96	27–97	
**MEDICAL HISTORY**, ***n*** **(%)**				
Cholestasis	2 (1.1)	1 (4.0)	1 (0.6)	0.25
Diabetes	16 (8.7)	4 (16.0)	12 (7.7)	0.24
Dysthyroidism	20 (11.1)	7 (28.0)	13 (8.3)	0.009^*^
Renal failure	8 (4.4)	3 (12.0)	5 (3.2)	0.08
Anxiety	7 (3.8)	1 (4.0)	6 (3.8)	1
Depression	16 (8.8)	4 (16.0)	12 (7.7)	0.24
Haemopathy	6 (3.3)	0	6 (3.8)	1
Skin diseases	16 (8.8)	4 (16.0)	12 (7.7)	0.24
Vitiligo	6 (3.3)	4 (16.0)	2 (1.3)	0.003^*^
Psoriasis	4 (2.2)	0	4 (2.6)	1
Eczema	2 (1.1)	0	2 (1.3)	1
Lichen	1 (0.5)	0	1 (0.6)	1
Rosacea	1 (0.5)	0	1 (0.6)	1
Caustic dermatitis	1 (0.5)	0	1 (0.6)	1
Pityriasis rubra pilaris	1 (0.5)	0	1 (0.6)	1
**HISTOLOGICAL TYPE**, ***n*** **(%)**				
Melanoma	175 (96.7)	23 (92.0)	152 (97.4)	0.19
SSM	53 (30.3)	11 (44.0)	42 (26.9)	0.67
MLM	13 (7.4)	0	13 (8.3)	0.21
Nodular	31 (17.7)	6 (24.0)	25 (16.0)	0.38
ALM	5 (2.8)	0	5 (3.2)	1
LM	7 (4.0)	1 (4.0)	6 (3.8)	1
Choroidal	5 (2.8)	0	5 (3.2)	1
Desmoplastic	2 (1.1)	0	2 (1.3)	1
Spitzoid	1 (0.5)	1 (4.0)	0	0.13
MD	60 (37.2)	4 (16.0)	56 (35.9)	0.04
Squamous cell carcinoma	6 (3.3)	2 (8.0)	4 (2.6)	0.20
**TUMOR CHARACTERISTICS**, ***n*** **(%)**				
Presence of ulceration	52 (28.7)	6 (24)	46 (29.5)	0.64
Breslow	4.9	4.2	5.0	
Mean				
Median	3.6	4.0	3.5	
Range	0–40	0.15–12	0–40	
Sentinel node Positive	8 (4.4)	1 (4.0)	7 (4.5)	1
Negative	5 (2.7)	1 (4.0)	4 (2.6)	0.52
Unmade	170 (93.9)	23 (92.0)	147 (94.2)	0.65
**LOCATION**, ***n*** **(%)**				
Head	35 (19.3)	3 (12.0)	32 (20.5)	0.41
Trunk	35 (19.3)	7 (28.0)	28 (17.9)	0.27
Lower limbs	47 (25.9)	7 (28.0)	40 (25.6)	0.80
Upper limbs	25 (14.3)	5 (20.0)	20 (12.8)	0.33
Unknown	24 (13.2)	2 (8.0)	22 (14.1)	0.54
Neck	4 (2.2)	1 (4.0)	3 (1.9)	0.45
MD	11 (6.1)	0	11 (7.1)	0.36
**METASTASIS**, ***n*** **(%)**				
Site number ≥3	110 (60.7)	13 (52.0)	97 (62.2)	0.26
<3	63 (35.3)	12 (48.0)	51 (32.7)	
Brain metastasis	35 (19.3)	5 (20.0)	30 (19.2)	1
Hepatic metastasis	46 (25.4)	1 (4.0)	45 (28.8)	0.06
**MUTATION STATUS (FOR MELANOMA)**, ***n*** **(%)**				
BRAF	44 (25.1)	6 (26.0)	38 (24.3)	1
NRAS	38 (21.7)	7 (26.1)	31 (19.9)	0.43
CKIT	6 (3.4)	0	6 (3.8)	1
MD and lack of mutation	87 (49.7)	10 (43.0)	77 (49.3)	0.39

*MD, missing data*.

* *p ≤ 0.05*.

Pembrolizumab (44.7%), nivolumab (35.9%), ipilimumab (17.1%), and cemiplimab (2.3%) were prescribed as first-line treatments. The average number of infusions was 13.6; the median was 7, and the extreme values were 1–86 infusions. Thirty-six patients (19.8%) received a second line of ICIs: ipilimumab for 19, nivolumab for 10, pembrolizumab for 7, and cemiplimab for 1. The average number of second-line infusions was 5.1, with a median of 4 and extreme values of 1–36. Only six patients were treated with third-line ICIs.

Eighty-one patients (44.7%) received treatment prior to treatment with immunotherapy, including radiotherapy (38.3% of patients), chemotherapy (33.3%), targeted therapy (27.2%), and interferon alpha (18.5%). Other therapies were previously used in eight patients: cetuximab in 3, ipilimumab in 1, imiquimod in 1, masitinib in 1, and nilotinib in 1. One patient was under a research protocol and received either nivolumab or ipilimumab.

### ICIs Associated With Pruritus

After the introduction of ICIs, pruritus not related to any skin disease was reported by 25 patients (13.8%); there were cases of 23 melanoma and two of squamous cell carcinoma. According to data presented in Table 2, pruritus occurred in 15 patients (60.0%) treated with pembrolizumab (in one of them, as a 2nd-line treatment after ipilimumab), in six patients (24.0%) treated with nivolumab, in two patients (8.0%) treated with ipilimumab (in one of them, as a 2nd-line treatment after pembrolizumab) and in two patients (8.0%) treated with cemiplimab. The average number of infusions was 25.4, the median was 21, and the extreme values were 3–81 infusions. Pruritus appeared after a mean of 8.1 months after ICIs were prescribed (median 6 months, range 0–25, 7.5 of standard deviation). On average, pruritus appeared after 12.2 infusions (median 6 and range 1–48).

**Table 2:** Treatment at the onset of pruritus.

**N^**°**^ patient**	**First line ICI's**	**Starting date (MM/YY)**	**Closing date**	**2nd line**	**Starting**	**Closing**	**Occurrence of pruritus**	**Occurrence in months**	**Regression**	**In days**	**Pruritus at last news**
1	Nivolumab	09.17	10.19				07.19	22	Yes	21	No
2	Ipilimumab	03.13	06.14	Pembrolizumab	10.14	04.15	04.15	25	Yes	10	No
3	Prembrolizumab	11.15	01.17				04.16	5	Yes	252	No
4	Pembrolizumab	11.16	08.17				02.17	8	Yes	MD	No
5	Pembrolizumab	09.17	02.18	Ipilimumab	02.18	05.18	03.18	6	Yes	56	No
6	Nivolumab	01.17	10.17	Pembrolizumab	11.17	03.18	04.17	3	Yes	28	No
7	Pembrolizumab	01.19	09.19				08.19	7	No		Yes
8	Pembrolizumab	11.15	11.17				09.17	22	Yes	MD	No
9	Nivolumab	01.18	10.18				01.18	0	Yes	280	No
10	Pembrolizumab	07.17	11.17	Ipilimumab	12.17	02.18	10.17	3	Yes	21	No
11	Pembrolizumab	07.15	09.16				02.16	7	Yes	56	No
12	Pembrolizumab	03.17	09.19				12.17	9	Yes	14	No
13	Pembrolizumab	07.17	01.19				03.18	8	Yes	280	No
14	Pembrolizumab	11.15	08.16				02.16	3	Yes	42	No
15	Nivolumab	06.17	11.17	Nivolumab	01.19	05.19	08.17	2	Yes	84	No
16	Cemiplimab	08.18	09.19				10.18	2	Yes	MD	No
17	Pembrolizumab	02.18	10.19				06.18	4	No		Yes
18	Nivolumab	02.16	09.19				05.17	3	Yes	28	No
19	Pembrolizumab	09.17	07.19				05.19	20	Yes	15	No
20	Cemiplimab	08.18	09.19				03.19	7	No		Yes
21	Pembrolizumab	04.18	09.19				05.18	1	Yes	15	No
22	Pembrolizumab	02.18	10.19				04.19	14	Yes	112	No
23	Pembrolizumab	01.19	10.19				04.19	3	Yes	140	No
24	Ipilimumab	09.14	11.14	Pembrolizumab	01.18	06.18	10.14	1	Yes	28	No
25	Nivolumab	06.16	08.18				12.17	18	Yes	28	No

*MD, missing data; dates are expressed in months/years. Occurrence in months: the number of months after the onset of pruritus after the start of immunotherapy. Regression: regression of pruritus in days and the persistence of the pruritus at the last follow-up*.

### Characteristics and Evolution of Pruritus

Among the 25 patients, pruritus was localized in 13 (52.0%) and was diffuse in the others. When localized, the involved areas were the trunk (nine cases, 36.0%), the upper limbs (seven cases, 28.0%), the lower limbs (six cases, 24.0%) and the neck (one case, 4.0%) (some patients had pruritus in several areas). No data on localization in seven patients were provided.

The treatment prescribed for pruritus was available in 14 cases: emollients for seven patients, antihistamines for seven patients and topical steroids for four patients. Twenty-two patients (88.0%) experienced regression of pruritus, among whom four experienced regression after treatment interruption and one experienced regression in a mean time of 79.4 days (from 10 to 280 days).

### Pruritus and Other Adverse Events

Patients presented with a large variety of adverse events.

Skin disease was described in 39 (21.5%) cases: vitiligo (20 patients, 11.0%), eczema (16 patients, 8.8%), maculopapular exanthema (eight patients, 4.4%), psoriasis (six patients, 3.3%), lichen (five patients, 2.7%), folliculitis (two patients, 1.1%), and urticaria (one patient, 0.5%). For patients with pruritus we found the following associated skin adverse events (Table 3): vitiligo (six patients, 24%), eczema (four patients, 16%), 0 maculopapular exanthema, psoriasis (one patient, 4.0%), lichen (one patient, 4.0%), folliculitis (one patient, 0.4%), and urticaria (one patient, 4.0%). We find a significant difference in the occurrence of vitiligo when comparing the two groups. The extra-cutaneous side effects observed are reported in Table 4.

**Table 3:** Cutaneous adverse events.

	**All patients, *n* (%) (*n* = 181)**	**Patients with pruritus, *n* (%) (*n* = 25)**	**Patients without pruritus (*n* = 156)**	***p***
Vitiligo	20 (11.1)	6 (24.0)	14 (8.9)	0.03
Eczema	16 (8.3)	4 (16.0)	12 (7.7)	0.24
Maculopapular exanthema	8 (4.4)	0	8 (5.1)	0.60
Psoriasis	6 (3.3)	1 (4.0)	5 (3.2)	0.59
Lichen/lichenoid	5 (2.7)	1 (4.0)	4 (2.7)	0.52
Folliculitis	2 (1.1)	1 (4.0)	1 (0.6)	0.25
Severe toxidermia	1 (0.5)	0	1 (0.6)	1
Urticaria	1 (0.5)	1 (4.0)	0	0.13

**Table 4:** Extra-cutaneous adverse events.

	**All patients, *n* (%) (*n* = 181)**	**Patients with pruritus, *n* (%) (*n* = 25)**	**Patients without pruritus (*n* = 156)**	***p***
**ENDOCRINE**				
Hypothyroidism	28 (15.5)	6 (24.0)	22 (14.1)	0.23
Hyperthyroidism	15 (8.3)	3 (12.0)	12 (7.7)	0.44
Hypophysitis	4 (2.2)	0	4 (2.6)	1
Diabetes	2 (1.1)	0	2 (1.3)	1
Adrenal insufficiency	1 (0.5)	0	1 (0.6)	1
**PULMONARY**				
Pneumopathy	1 (0.5)	0	1 (0.6)	1
Sarcoidosis	1 (0.5)	0	1 (0.6)	1
**DIGESTIVE**				
Cholestasis	52 (28.7)	8 (32.0)	44 (28.2)	0.81
Cytolysis	43 (23.7)	10 (40.0)	33 (21.1)	0.07
Colitis	10 (5.5)	2 (8.0)	8 (5.1)	0.63
Gastritis	2 (1.1)	0	2 (1.3)	1
Pancreatitis	1 (0.5)	0	1 (0.6)	1
**RENAL**				
Renal failure	10 (5.5)	4 (16.0)	6 (3.8)	0.03^*^
Nephropathy	2 (1.1)	0	2 (1.3)	1
**MUSCULOSKELETAL**				
Arthralgia	17 (9.4)	3 (12.0)	14 (8.9)	0.71
Myalgia	4 (2.2)	0	4 (2.6)	1
Neuropathy	3 (1.7)	0	3 (1.9)	1
**OTHERS**				
Hypereosinophilia	20 (11.05)	9 (36.0)	11 (7.0)	0.0002^*^
Dry syndrome	8 (4.4)	3 (12.0)	5 (3.2)	0.08
Uveitis	2 (0.5)	0	2 (1.3)	1

* *p ≤ 0.05*.

Among these adverse events are those known to be pruritus inducers. Among the 25 patients who developed pruritus under immunotherapy, many were diagnosed with putative causes of pruritus (Table 4). In spite of our small numbers of patients we found, for immuno-induced effects, statistically significant differences for renal failure and hypereosinophilia.

In 6/25 patients, no more specific cause of pruritus was found at the onset of pruritus or in their backgrounds, other than ICI use. In 19/25 patients, other putative causes of pruritus were found. ICI-related adverse events that are known to be putative causes of pruritus were diagnosed before the onset of pruritus or concomitantly with the onset of pruritus: four cases of xerosis, two cases of chronic renal failure [one grade I and one grade II of the CTCAEv4 (Common Terminology Criteria for Adverse Events) classification], three cases of dysthyroidism, and two cases of cholestasis (two grade III of the CTCAEv4 classification) but no diabetes. For 10 cases, drugs (other than ICIs) that could also induce pruritus were noted. For drug causes we have selected two classes known to be responsible for pruritus. The two classes selected were antihypertensive, the class of enzyme conversion inhibitors and opiates. Last, five patients had a history of conditions that may induce pruritus: two cases of dysthyroidism, two of depression, one of diabetes, and one of vitiligo. However, these patients did not have pruritus before the introduction of ICIs and developed pruritus only after using these drugs.

### Survival Analysis

Tables 5, 6 show the results of survival analyses. Adjustment with the following covariates was performed: sex, presence or absence of cerebral metastases, and presence or absence of hepatic metastases. There was a statistically significant association between the presence of cerebral or hepatic metastases and the occurrence of pruritus. There was a non-significant risk ratio for survival based on the occurrence of pruritus (HR = 0.47, 95%CI: 0.19–1.18, *p*-value = 0.11). We did not find significant risk ratios in the specific cases of pruritus directly related to ICIs (HR = 0.70, 95% CI: 0.17–2.89 and HR = 0.38, 95% CI: 0.12–1.23, respectively).

**Table 5:** Survival analysis based on the appearance of pruritus after adjustment with covariates (sex, cerebral, and hepatic metastases).

**Variable**		**Hazard Ratio (95%CI)**	***p*-Value**
Sex	Male	1.06 (0.70, 1.59)	0.79
	Female	1	
Brain metastases	Yes	1.74 (1.07, 2.83)	0.025
	No	1	
Hepatic metastases	Yes	2.34 (1.53, 3.57)	<0.0001
	No	1	
Pruritus appearance	Yes	0.47 (0.19, 1.18)	0.11
	No	1	

*The HR for each variable corresponds to adjusted analysis for the other variables*.

**Table 6:** Survival analysis according to the appearance of different pruritus subtypes after adjustment with covariates (sex, cerebral, and hepatic metastases).

**Variable**		**Hazard Ratio (95%CI)**	***p*-Value**
Sex	Male	1.07 (0.71, 1.60)	0.76
	Female	1	
Brain metastases	Yes	1.76 (1.08, 2.86)	0.023
	No	1	
Hepatic metastases	Yes	2.35 (1.54, 3.60)	<0.0001
	No	1	
Pruritus appearance	Induced	0.70 (0.17, 2.89)	0.62
	Systemic	0.38 (0.12, 1.23)	0.11
	None	1	

*The HR for each variable corresponds to adjusted analysis for the other variables*.

## Discussion

In our population, we found that 13.8% of patients developed pruritus, which corresponds to data in the literature. For example, Sibaud et al. (24) reported an incidence of 13–20%, sometimes more frequent with ipilimumab or in combination, up to 47% (25). A higher frequency of pruritus was previously reported with ipilimumab than anti-PD1. In addition, Sibaud reported a global frequency of 24.5–35.5% with ipilimumab vs. 14–21 and 17–19% with pembrolizumab and nivolumab (24), and Phillips et al. found that the trunk and limbs were more often affected than the neck. Finally, a meta-analysis evaluating the frequency of pruritus reported 26.8% in patients receiving ipilimumab, 22.4% in those receiving nivolumab, and 21.4% in those receiving pembrolizumab. When these treatments were associated, the frequency was more than 40% (26).

The main finding of our study is that causes of pruritus other than ICIs may be detected in the majority of patients. Consequently, there is a need to be cautious before considering that pruritus is a side effect of ICIs and, furthermore, to stop use of ICIs because of its development. Nonetheless, no other putative cause of pruritus was found in a quarter of patients, which is a strong argument for considering that ICIs may be the direct cause of pruritus in these patients. Finally, ICIs might also induce pruritus through side effects, such as dermatological diseases, xerosis, renal failure, cholestasis, and dysthyroidism.

There is no known role of CTLA-4 nor PD-1 in pruritus and the mechanisms of these side effects need to be elucidated.

Pruritus associated with ipilimumab is believed to be a direct result of inhibiting CTLA4 and the resulting enhanced immune system activation in the skin through amplified T cell recognition of self-antigens (14, 18). In a previous study, 14% of patients with stage IV melanoma who received ipilimumab developed pruritic skin eruptions, with CTLA4 blockade believed to be the primary cause in eight of these patients (27). Notably, these patients presented superficial, perivascular CD4^+^-predominant T cell infiltrates with eosinophils in the dermis upon histological examination. Thus, the same pathways responsible for pruritus may also represent those involved in slowing tumor growth and increasing patient survival (18). Presumably, similar mechanisms should occur for anti-PD1 (28).

In our study, pruritus was not a rapidly occurring adverse event, appearing on average after 8.1 months and 12.2 infusions. According to Phillips, an average of 11 therapy cycles occurred before presentation in patients with pruritus only (17). Wang explained that a delayed onset of cutaneous side effects (more than 3 months) is common and that they can also occur after treatment discontinuation (29). This delay may be consistent with the presumed physiopathology of pruritus in immunotherapy.

The majority of patients experienced a regression of pruritus in our study (88%) but after cessation of treatment in only four. Most studies unfortunately do not detail the exact evolution of pruritus; nonetheless, management and therapeutic care are often described. However, this is not the case in our study because data on the management, in particular the duration of the treatments, were not necessarily available.

In our study, we found adverse events secondary to ICIs other than pruritus. Our findings involve higher numbers than in the study of Baxi, who found, for example, an incidence of 5.6% for hypothyroidism, 2.2% for pneumonitis, 0.7% for colitis, 0.2% for hepatitis, and 0.3% for hypophysitis in a total of 3,803 patients treated with anti-PD1 drugs, with any grade of severity combined (9). Indeed, our data showed 15.5% hypothyroidism, 5.5% colitis, 28.7 and 23.7% for cholestasis and cytolysis, 0.5% pneumonitis, and 2.2% hypophysitis. For cholestasis and cytolysis we took into account any increase from the high standard of ALAT, ASAT, GGT, PAL, and we did not use the CTCAE4 grade because it was not always indicated. Dermatologic IRAEs are the most frequent IRAEs. The safety profile of 10 mg/kg ipilimumab across phase II trials was associated with 47% skin IRAEs vs. 39% gastrointestinal IRAEs, 3% hepatitis, and 4% hypophysitis (10).

Skin toxicities of ICIs are variable: rash, pruritus, lichenoid dermatitis, psoriasis, vitiligo, auto-immune dermatoses, alopecia, and nail involvement, to list only the main ones (24). We also found a variety of cutaneous immune-related adverse events. The most frequent were vitiligo (11%), followed by eczema (8.3%), maculopapular exanthema (4.4%), and lichenoid dermatitis (only 2.7%). Some data in the literature show a higher rate of lichenoid dermatosis compared to our study, e.g., 17% for Boada (8). This difference can be explained by a difficulty sometimes in clinically classifying patients in the absence of histological confirmation. Different authors seem to agree that these toxicities are rarer and less severe with anti-PD1 than with ipilimumab (7).

Several authors report an association between survival and the occurrence of side effects. Eggermont evaluated the association between these factors and survival for patients with stage III melanoma treated with pembrolizumab in a double-blind randomized clinical trial comparing pembrolizumab and placebo. In the pembrolizumab arm, he found a reduction in the risk of recurrence of death after the onset of side effects (19). Chan et al. explained that dermatitis may be a sign of increased immune activation and therefore a better anti-tumor response in relation to lymphocyte infiltration. Patients who had one or more cutaneous reactions (eczema, lichenoid reactions or vitiligo-like depigmentation) were approximately half as likely to experience disease progression than patients who did not develop those IRAEs (23). In a prospective observational study, Hua reported a better tumor response in patients with vitiligo than in patients who did not develop vitiligo while on pembrolizumab (22). This suggests that the onset of vitiligo is an IRAE associated with a clinical benefit. Freeman-Keller rash and vitiligo were associated with a statistically significant overall survival difference for patients with resected and unresectable metastatic melanoma treated with nivolumab (20).

Our survival analysis did not find an association between pruritus and better survival. This might be related to the heterogeneity of tumor characteristics. Moreover, we were not able to demonstrate better survival in the population who developed pruritus without any other detected putative cause of pruritus than ICIs. Our results, although not significant, are consistent with those previously reported for other undesirable skin effects (23).

Our study had some limitations. The main limitation is that it is a monocentric and retrospective study; when files are analyzed retrospectively, data are inevitably missing, or the information is incomplete. Our results warrant further investigation in a prospective study, procuring more information on the characteristics of pruritus (notably on its severity and consequences on quality of life) and the treatment of patients. Skin biopsies could also permit analysis of histological changes in the skin as well as small-fiber neuropathies. Another limitation is the relatively small size of our sample.

In conclusion, our results indicate that pruritus can be a direct side effect of ICIs but may also be an indirect side effect or due to a concomitant disorder or treatment without any relationship to ICIs. Further studies would allow a better understanding of these three pathophysiological pathways. The occurrence of pruritus might be associated with better survival, and studies with larger sample sizes should be able to confirm this trend.

## Data Availability Statement

The original contributions presented in the study are included in the article/supplementary material, further inquiries can be directed to the corresponding author/s.

## Ethics Statement

The studies involving human participants were reviewed and approved by ethics committee of Brest (B2019CE.19). The patients/participants provided their written informed consent to participate in this study.

## Author Contributions

NS: research work and writing article. EN: participation in the preparation of statistical analyses. DL, ME, and MF: supervision and coaching. EB and LM: supervision and coaching, and proofreading work. All authors contributed to the article and approved the submitted version.

## Conflict of Interest

DL: BMS, MSD, Novartis, Pierre Fabre. LM: BMS, Sanofi. The remaining authors declare that the research was conducted in the absence of any commercial or financial relationships that could be construed as a potential conflict of interest.
